# Splenic vein resection together with the pancreatic parenchyma versus separated resection after isolation of the parenchyma during distal pancreatectomy (COSMOS-DP trial): study protocol for a randomised controlled trial

**DOI:** 10.1186/s13063-018-2756-7

**Published:** 2018-07-11

**Authors:** Suguru Yamada, Tsutomu Fujii, Manabu Kawai, Toshio Shimokawa, Masafumi Nakamura, Yoshiaki Murakami, Sohei Satoi, Hidetoshi Eguchi, Yuichi Nagakawa, Yasuhiro Kodera, Hiroki Yamaue

**Affiliations:** 1 Department of Gastroenterological Surgery (Surgery II), Nagoya University Graduate School of Medicine, Nagoya, Japan; 2 Department of Surgery and Science, Graduate School of Medicine and Pharmaceutical Sciences, University of Toyama, Toyama, Japan; 3 Second Department of Surgery, Wakayama Medical University, Kimiidera, Wakayama, 641-8510 Japan; 4 Clinical Study Support Centre, Wakayama Medical University, Wakayama, Japan; 5 Department of Surgery and Oncology, Graduate School of Medical Sciences, Kyushu University, Fukuoka, Japan; 6 Department of Surgery, Institute of Biomedical and Health Sciences, Hiroshima University, Hiroshima, Japan; 7 Department of Surgery, Kansai Medical University, Hirakata, Japan; 8 Department of Gastroenterological Surgery, Osaka University Graduate School of Medicine, Osaka, Japan; 9 Department of Surgery, Tokyo Medical University, Tokyo, Japan

**Keywords:** Distal pancreatectomy, Splenic vein, Intra-abdominal haemorrhage, Pancreatic fistula, Mechanical stapler

## Abstract

**Background:**

In distal pancreatectomy (DP), it is customary to ligate and divide the splenic vein after isolating it from the pancreatic parenchyma. This is considered essential to prevent disruption of the stump of the splenic vein and consequent intra-abdominal haemorrhage in the event of pancreatic fistula (PF). However, this procedure can be technically demanding, especially when the vein is firmly embedded in the pancreatic parenchyma. The objective of the COSMOS-DP trial is to confirm the non-inferiority of resection of the splenic vein embedded in the pancreatic parenchyma compared with the conventional technique of isolating the splenic vein before resection during DP using a mechanical stapler.

**Methods:**

Patients with diseases of the pancreatic body and tail whose pancreatic parenchyma and splenic vein can be divided concurrently during open or laparoscopic DP are considered eligible for inclusion. This study is designed as a multicentre prospective randomised phase III trial. Eligible patients will be centrally randomised to either Arm A (resection of the splenic vein after isolation from the pancreatic parenchyma) or Arm B (co-resection of the vein together with the pancreas). This study aims to establish the non-inferiority of the safety of Arm B compared with that of Arm A; the primary endpoint is the incidence of PF (ISGPF grade B/C).

**Discussion:**

The COSMOS-DP trial will establish the safety of this procedure, such that it can be recommended with more confidence. The use of this procedure will likely result in significant reductions in operative time and blood loss during DP.

**Trial registration:**

ClinicalTrials.gov, NCT02871804. Registered on 27 July 2016.

**Electronic supplementary material:**

The online version of this article (10.1186/s13063-018-2756-7) contains supplementary material, which is available to authorized users.

## Background

In general, distal pancreatectomy (DP) involves not only mandatory dissection of the pancreas but also dissection of the splenic artery and vein. During this surgical procedure, the splenic vein is isolated from the pancreatic parenchyma before being ligated and divided. The reason for this is to prevent intra-abdominal haemorrhage from the stump of the splenic vein following the occurrence of pancreatic fistula (PF), which is commonly observed after DP (8.6–42.3%) [1–8]. Although it is relatively easy to isolate the splenic vein at the confluence of the portal and splenic veins, it is often firmly embedded in the pancreatic parenchyma at more distal regions of the pancreas. Furthermore, the splenic vein can be difficult and time-consuming to isolate due to the necessity of carefully handling all small branches that flow from the parenchyma to the splenic vein. Thus, at times, this procedure can lead to hardships, as well as an unexpectedly large volume of blood loss.

More recently, mechanical staplers have been increasingly used to dissect the pancreas, particularly when DP is performed using a laparoscopic approach. Under such circumstances, the splenic vein is often dissected together with the pancreatic parenchyma, with no attempt of isolation. This method of pancreatic dissection has become the standard at some institutions and has been reputed for its apparent technical simplicity [9, 10]. However, PF occurring after this type of resection is of deep concern to surgeons due to the risk of intra-abdominal bleeding from the stump of the splenic vein, which could then be immersed in effusion rich in pancreatic juice.

To date, no scientific investigation of the safety of this useful but potentially hazardous surgical procedure has been performed. Therefore, we plan to conduct a prospective randomised study to establish the safety of this procedure so that it can be recommended with more confidence. The use of this procedure will likely result in significant reductions in operative time and blood loss during DP.

## Methods/Design

### Aim

The aim of the COSMOS-DP trial is to establish the non-inferiority of the safety of resecting the splenic vein together with the pancreatic parenchyma compared with that of the conventional technique of isolating the vein from the pancreas before ligation and division during DP using mechanical staplers.

### Study population

Patients undergoing open or laparoscopic DP for pancreatic body and tail cancer, intra-ductal papillary mucinous neoplasm, neuroendocrine tumours, mucinous cystic neoplasm, or metastatic pancreatic tumours or similar are eligible for inclusion in this study. In addition, simultaneous resection of the pancreatic parenchyma and splenic vein in one session will be rendered possible through the evaluation of preoperative imaging study findings. Patients indicated for spleen-preserving distal pancreatectomy (SPDP) will be excluded. The Warshaw operation (spleen-preserving and splenic artery/vein resection) will be included. The extent of lymph node dissection and whether the celiac axis is dissected will be left to the discretion of the surgeons. A detailed overview of all eligibility criteria is provided in Table 1.

**Table 1:** Eligibility criteria

Inclusion criteria	Exclusion criteria
(1) Elective open or laparoscopic DP for diseases of the pancreatic body and tail (2) ECOG performance status (PS) = 0–1 (3) Age ≥ 20 years (4) Maintenance of function of the major organs (bone marrow, liver, kidney, lung, etc.) (a) White blood cells ≥ 2500/mm^3^ (b) Haemoglobin ≥ 9.0 g/dL (c) Platelets ≥ 100,000/mm^3^ (d) Total bilirubin ≤ 2.0 mg/dL (e) Creatinine ≤ 2.0 mg/dL (5) Sufficient understanding of the study to provide written informed consent	(1) Splenic vein-preserving DP (2) Superior mesenteric vein or portal vein invasion (3) Pancreatic trauma (4) Preoperative inflammatory pancreatic disease (pancreatitis) (5) Requirement of anti-coagulant treatment during or after surgery^a^ (6) Severe ischemic cardiovascular disease (7) Liver cirrhosis or active hepatitis (8) Need for oxygen due to interstitial pneumonia or lung fibrosis (9) Dialysis due to chronic renal failure (10) Need for surrounding organ resection (stomach, colon, etc.), excluding the left adrenal gland and gall bladder (11) Active multiple cancer that is thought to influence the occurrence of adverse events (12) Difficulty with study participation due to psychotic disease or symptoms (13) When a surgeon considers the use of stapler as inappropriate (14) Inappropriate for the study objectives

^a^Anti-coagulant treatment at 24 h after surgery is allowed

DP distal pancreatectomy

### Study design

**Fig. 1:**
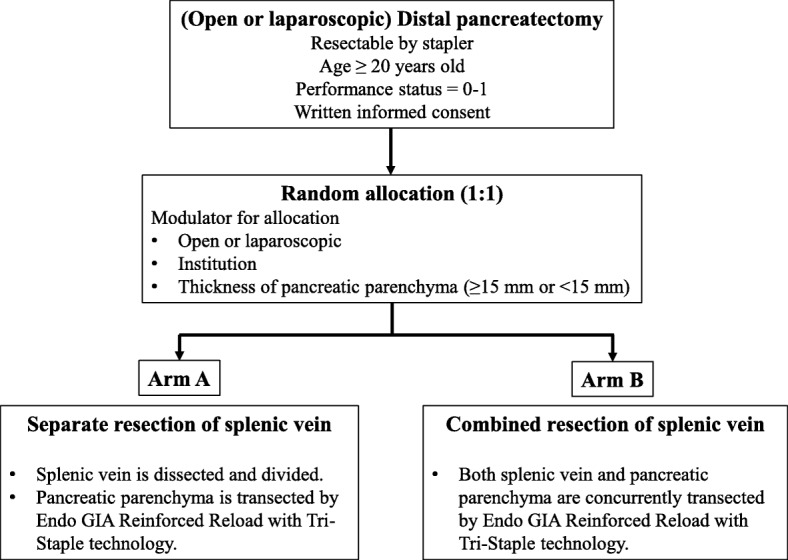
Flow diagram of COSMOS-DP trial

This study is designed as a multicentre (45 institutes, Additional file 1: Table S1, Institution list) prospective randomised phase III trial; central randomisation and registration system will be applied (1:1). After assessing the patients for eligibility, they will be centrally randomised to either Arm A (separate resection of the splenic vein) or Arm B (combined resection of the splenic vein). Upon randomisation, the patients will be stratified by the surgical approach used (open or laparoscopic), institution and thickness of the pancreatic parenchyma (< 15 mm or ≥ 15 mm) [11], but not for the consistency of the pancreas. However, the consistency will be reported as one of the key variables in the case report form. We will use Pcock and Simon’s minimization method for random assignments and Mersenne Twister for random number generation (Fig. 1, flow diagram of the COSMOS-DP trial).

The primary endpoint is the incidence of PF grade B/C. The secondary endpoints will be outcome measures related to surgery, such as the operative time, volume of blood loss, preoperative thickness of the resected pancreatic parenchyma, haemostasis of the staple line, integrity of the staple line, incidence of pancreatic injury, need for additional sutures to securely close the pancreatic stump, duration of drainage tube placement, postoperative hospital stay duration, and incidence of conversion from laparoscopic surgery to open surgery. The outcome measures related to complications include the incidence of PFs of all grades, incidence of grade C PF, incidence of intra-abdominal haemorrhage, incidence of all complications, comparison of the thickness of the resected pancreatic parenchyma with the incidence of PF grade B/C, mortality, and incidence of thrombosis of the splenic vein (at one and six months after surgery).

This study was designed to prove the non-inferiority of Arm B compared with Arm A in terms of the primary endpoint [12].

### Statistical analysis

This trial was designed to evaluate the non-inferiority of group B compared with group A in terms of the incidence of PF grade B/C. The incidence of clinically relevant PF (grade B/C) after DP using a stapler has been reported to be 1.9–20.3% in recent clinical trials [1, 2, 5, 13]. Therefore, for an assumed PF incidence rate of 10% with a non-inferiority margin of 9%, the difference of allowable PF incidence rate between groups A and B is 0.09, and the allowable odds ratio is 2.11. When the statistical analysis is performed for a significance level of α = 0.05 (one side) in a non-inferiority design, 138 patients are calculated to be required per arm, with a power 100 (1-β) of > 80%, under the assumption that a small number of patients may be deemed ineligible and may thus be excluded from the analysis. Furthermore, as approximately 5% of the patients are expected to be ineligible for surgery as indicated by the laparotomy or laparoscopic findings, the sample size was eventually increased to 304 patients (152 patients per arm).

As secondary endpoints, we will compare binary variables with Fisher’s exact test, continuous variables with the Mann–Whitney U test, and survival outcomes with the log-rank test. All results will be analysed using the full analysis set (FAS), which will include all patients except for those deemed ineligible after registration.

### Interventions

#### Surgical resection

Before pancreatic transection, concurrent division of the splenic artery using a mechanical stapler will not be permitted. A linear stapler, Endo GIA Reinforced Reload with Tri-Staple Technology (Black Cartridge, Covidien®), will be used in all patients. The pancreatic parenchyma will be compressed with the stapler at the planned line of resection for > 5 min before transection is performed. For the patients in Arm A, the splenic vein will be isolated from the pancreatic parenchyma and dissected after ligation. For those in Arm B, the splenic vein will be transected concurrently with the pancreatic parenchyma using the aforementioned stapler. Anti-coagulant treatment, such as low-molecular-weight heparin or fondaparinux, will be permitted at 48 h after surgery.

### Intra-abdominal drainage

To evaluate postoperative PF, which is the primary endpoint, drainage tubes will be placed before closure of the abdomen. The number and sites of the drainage tubes that are inserted will be recorded on the data sheet. The timing of removal of the drainage tubes will also be specified.

### Intraoperative photography

To confirm that the surgical procedures are conducted as allocated at the time of central judgement, two photographs (before and after pancreatic transection) will be taken for all patients. Central judgement will be conducted biannually for all of the registered patients. At that time, the photographs will be reviewed by more than two members of the committee.

### Concurrent and supportive treatments

Antibiotics, plasma expanders, blood products, analgesic drugs, H2 blockers and proton pump inhibitors will be used for intra- and postoperative management at the discretion of the surgeons. In addition, there are no regulations on the drugs used to control complications and adverse events. Prophylactic administration of octreotide will not be permitted.

### Chemotherapy and radiotherapy

There are no regulations regarding the use of preoperative, intraoperative or postoperative chemotherapy or radiotherapy as treatments in this study. However, details including the treatment regimen and cycles given of the preoperative treatment are to be reported in the case report form.

### Follow-up after surgery

**Fig. 2:**
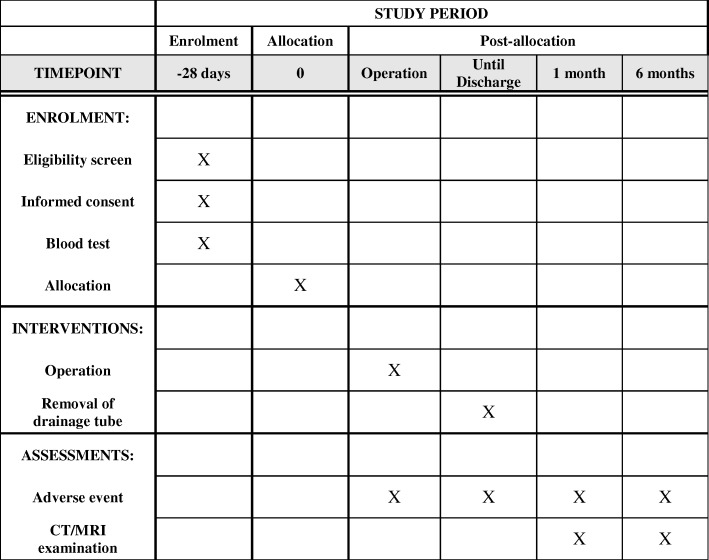
Study calendar

The presence of thrombus in the splenic vein will be evaluated at one and six months after surgery by enhanced computed tomography or magnetic resonance imaging (Fig. 2, study calendar).

### Interim analysis and monitoring

Interim analysis will be performed once, taking multiplicity into account using the Lan–DeMets method with O’Brien and Fleming type boundaries. The Data and Safety Monitoring Committee will independently review the interim analysis report and stop the trial early if necessary.

Central monitoring will be performed each year by the data centre to evaluate the study progress and ensure for study quality. The following aspects will be monitored: (1) data accumulation; (2) patient eligibility; (3) severe adverse events; (4) protocol deviations; (5) reasons for cessation or expiration of the protocol; (6) background factors of the patients; and (7) other problems concerning study progress and safety.

### Definition of postoperative complication

A complication is defined as an event occurring within six months after surgery.

### Pancreatic fistula

The definition of PF is based on the International Study Group of Postoperative Pancreatic Fistula (ISGPF) classification [12].

### Delayed gastric emptying

The definition of delayed gastric emptying is based on the International Study Group of Pancreatic Surgeons (ISGPS) classification [14].

### Intra-abdominal bleeding

The definition of intra-abdominal bleeding is based on the International Study Group of Pancreatic Surgeons (ISGPS) classification [14].

### Other complication

The definition of other postoperative complication is based on the Clavien-Dindo classification [15].

## Discussion

In DP, the pancreatic parenchyma and splenic vein are generally transected and resected separately. Most pancreatic surgeons believe that by performing this procedure, intra-abdominal haemorrhage from the stump of the splenic vein due to PF will be prevented. However, the recent development of mechanical staplers, particularly when DP is performed using a laparoscopic approach, enables transection of the splenic vein together with the pancreatic parenchyma. In fact, this method is safely performed at some institutions and the technical simplicity of the method has been reported [9, 10]. However, no clinical trial to demonstrate the safety of this useful surgical procedure has been performed.

In this clinical trial, it would be more reasonable to compare the incidence of intra-abdominal haemorrhage as a primary endpoint because of the above-mentioned background. However, this complication is expected to be extremely rare, particularly in DP. Thus, we will set the incidence of PF grade B/C as a primary endpoint and evaluate the incidence of intra-abdominal haemorrhage as a secondary endpoint in this study. In addition, this trial is designed to evaluate the non-inferiority of Arm B (combined resection of the splenic vein) compared with Arm A (separate resection of the splenic vein) in terms of the incidence of PF grade B/C.

Upon randomisation, the patients will be stratified by the surgical approach used (open or laparoscopic), institution and thickness of the pancreatic parenchyma (< 15 mm or ≥ 15 mm) [11]. The reason is that these factors may affect the incidence of PF grade B/C, which might impact the incidence of intra-abdominal haemorrhage. Therefore, we will set these three factors for allocation in this study for appropriate analysis and evaluation.

In this multicentre, prospective, randomised phase III trial (COSMOS-DP trial), we plan to establish the safety of this procedure, such that it can be recommended with more confidence. The use of this procedure will likely result in significant reductions in operative time and blood loss during DP (Additional file 2).

### Trial status

The COSMOS-DP trial was opened in August 2016. At the time of submission for this paper (May 2018), protocol version is ver.2.2. The completion date is estimated to be December 2019.

## Additional files

Additional file 1: Institution list. (DOCX 16 kb)

Additional file 2: SPIRIT 2013 Checklist: Recommended items to address in a clinical trial protocol and related documents. (DOC 121 kb)

## Abbreviations

DP: Distal pancreatectomy
FAS: Full analysis set
ISGPF: International Study Group of Postoperative Pancreatic Fistula
ISGPS: International Study Group of Pancreatic Surgeons
PF: Pancreatic fistula
SPDP: Spleen-preserving distal pancreatectomy
