# Supplementary material for: Splenic vein resection together with the pancreatic parenchyma versus separated resection after isolation of the parenchyma during distal pancreatectomy (COSMOS-DP trial): study protocol for a randomised controlled trial
Source: Trials. 2018 Jul 11;19:369. doi: 10.1186/s13063-018-2756-7 (PMC6042420; doi:10.1186/s13063-018-2756-7)
Supplement: Supplementary file 1 — Institution list. (DOCX 16 kb) [file 13063_2018_2756_MOESM1_ESM.docx]

| 1. Nagoya University Hospital |
| --- |
| 1. Wakayama Medical University Hospital |
| 1. Kumamoto University Hospital |
| 1. Kinki University Hospital |
| 1. Tohoku University Hospital |
| 1. National Defence Medical College Hospital |
| 1. Showa University Hospital |
| 1. Bantane Hotokukai Hospital |
| 1. Nara Medical University Hospital |
| 1. Keio University Hospital |
| 1. Toho University, Omori Hospital |
| 1. Kansai Medical University Hospital |
| 1. The Hospital of Hyogo College of Medicine |
| 1. Oita Red Cross Hospital |
| 1. Toyama Prefectural Central Hospital |
| 1. Fukuyama Medical Center |
| 1. Sendai Kousei Hospital |
| 1. Kyushu University Hospital |
| 1. Kyoto Katsura Hospital |
| 1. St. Marianna University School of Medicine Hospital |
| 1. Kansai Rosai Hospital |
| 1. Tokyo Medical And Dental University Hospital Faculty of Medicine |
| 1. University Hospital Kyoto Prefectural University of Medicine |
| 1. Osaka University Hospital |
| 1. Toyama University Hospital |
| 1. Hiroshima University Hospital |
| 1. Oita University Hospital |
| 1. Sapporo Medical University Hospital |
| 1. University of Miyazaki Hospital |
| 1. Gifu University Hospital |
| 1. Kyoto University Hospital |
| 1. Japanese Red Cross Otsu Hospital |
| 1. Fukushima Medical University Hospital |
| 1. Saiseikai Yokohamashi Tobu Hospital |
| 1. Osaka City University Hospital |
| 1. Hiroshima City Hiroshima Citizens Hospital |
| 1. Japanese Red Cross Musashino Hospital |
| 1. University of Yamanashi Hospital |
| 1. Hokkaido University Hospital |
| 1. Tokyo Medical University Hospital |
| 1. Teikyo University Medical Center |
| 1. Aichi Cancer Center |
| 1. Kurume University Hospital |
| 1. Yamaguchi University Hospital |
| 1. Osaka City General Hospital |
